# Addressing the unmet needs of bipolar disorder in Australia and beyond

**DOI:** 10.1177/00048674251361727

**Published:** 2025-09-12

**Authors:** Sue M Cotton, Melissa Hasty, Philip Mitchell, Greg Murray, Ian B Hickie, Patrick D McGorry, Ken Walder, Olivia M Dean, Cathy Mihalopoulos, Alison R Yung, Tamsyn E Van Rheenen, Andrew A Nierenberg, Jan Scott, Lana J Williams, Lesley Berk, Christopher G Davey, Jacob J Crouse, Elizabeth Scott, Frank Iorfino, Kate Filia, Mary Lou Chatterton, Craig Macneil, Tania Perich, Emma Morton, Aswin Ratheesh, Michael Berk

**Affiliations:** 1 School of Psychological Sciences, Faculty of Medicine, Nursing and Health Sciences, Monash University, Clayton, VIC, Australia; 2 Turner Institute for Brain and Mental Health, Clayton, VIC, Australia; 3 Centre for Youth Mental Health, The University of Melbourne, Parkville, VIC, Australia; 4 Orygen Ltd, Parkville, VIC, Australia; 5 Discipline of Psychiatry and Mental Health, Faculty of Medicine and Health, University of New South Wales, Randwick, NSW, Australia; 6 Centre for Mental Health and Brain Sciences, School of Health Sciences, Swinburne University, Melbourne, VIC, Australia; 7 Brain and Mind Centre, The University of Sydney, Camperdown, NSW, Australia; 8 Institute for Mental and Physical Health and Clinical Translation, School of Medicine, Deakin University, Geelong, VIC, Australia; 9 Florey Institute for Neuroscience and Mental Health, The University of Melbourne, Parkville, VIC, Australia; 10 Monash University Health Economics Group, School of Public Health and Preventive Medicine, Monash University, Melbourne, VIC, Australia; 11 Department of Psychiatry, The University of Melbourne, Parkville, VIC, Australia; 12 Harvard Medical School, Boston, MA, USA; 13 Dauten Family Center for Bipolar Treatment Innovation, Massachusetts General Hospital, Boston, MA, USA; 14 Academic Psychiatry, Institute of Neuroscience, Newcastle University, Newcastle, UK; 15 School of Psychology, Western Sydney University, Sydney, NSW, Australia; 16 Translational Health Research Institute, Western Sydney University, Sydney, NSW, Australia

**Keywords:** Bipolar disorder, mania, depression, early intervention, mental health, treatment, psychiatry, stakeholder engagement, capacity building

## Abstract

People impacted by bipolar disorder are confronted by many unmet needs that contribute to the overall burden associated with the disorder. We do not have a good understanding of the underlying pathology of bipolar disorder, so we do not have biomarkers to accurately identify those who are at risk of developing the disorder. Delayed diagnosis is the norm, and it can take a decade or more for an individual to receive a diagnosis and to start appropriate treatment. We have evidence-based treatments such as lithium and psychosocial therapies; however, their availability and use are limited. We need a consolidated approach to advance indicated prevention and early intervention for bipolar disorder. In this viewpoint article, we describe these barriers in detail as well as introduce international and national work that is being done to progress the field. At the national level, we introduce the National Health and Medical Research Council Centre for Research Excellence in Bipolar Disorder. The Centre for Research Excellence in Bipolar Disorder comprises a multidisciplinary team of experts from Australia and internationally who are working together to develop a better understanding of opportunities for indicated prevention and early intervention as well as to improve interventions for those impacted by the disorder. Here we describe our research framework, stakeholder engagement activities and strategies for workforce development and capacity building. Ultimately by working together we will attempt to address many of issues faced by individuals impacted by bipolar disorder.

In this Viewpoint article, we outline the need for research investment and service transformation to better meet the needs of people living with bipolar disorder (BD). We highlight international work as well as the role of the National Health and Medical Research Council (NHMRC) Centre of Research Excellence in Bipolar Disorder (CORE-BD) in identifying and implementing solutions for progressing indicated prevention and early intervention for BD.

## Why BD?

BD encompasses a spectrum of mood disorders characterised by fluctuations in mood, energy and activity levels (American Psychiatric Association, 2013; World Health Organization, 2004). Bipolar I Disorder is marked by the presence of at least one manic episode, often with depressive episodes. Bipolar II Disorder involves hypomanic and depressive episodes but no full manic episodes. These disorders pose significant individual, familial, social, medical and economic burdens (GBD 2019 Mental Disorders Collaborators, 2020; Kieling et al., 2024). Contributing to these burdens are functional impairments, diminished health-related quality of life (QoL) and premature mortality due to suicide or physical health comorbidities (Yocum et al., 2023). In 2019, BD cost the Australian Federal Government approximately $8.08 billion which equates to ~34% of health and welfare expenditure (Harper, 2020). Yet BD remains the most poorly supported and researched of any of the top 10 burdens of disability (Post, 2020; Torrey et al., 2021; Vieta and Angst, 2021; Zhong et al., 2024).

Despite more than a century of research, there remains much more to know about BD. Although familial risk and complex genetics play a role in BD (Gordovez and McMahon, 2020; Harrison et al., 2018; O’Connell et al., 2025), our understanding of its pathophysiology is limited (Mullins et al., 2021; O’Connell et al., 2025). It is difficult to predict who is at risk of developing BD (Brietzke et al., 2016; Rowland and Marwaha, 2018). Diagnostic delay is the norm; it can take a decade or more between symptom onset to diagnosis and treatment (Dagani et al., 2017; Keramatian et al., 2022; Scott et al., 2022). Factors contributing to diagnostic delays can be categorised according to issues associated with (1) our *diagnostic frameworks* (i.e. problems with Diagnostic and Statistical Manual of Mental Disorders and International Classification of Disease in terms of inconsistent criteria, arbitrary subtypes, issues with reliability and validity (Angst, 2013; Angst et al., 2020; Kaltenboeck et al., 2016; Kessing et al., 2021; Malhi et al., 2019)); (2) *differential diagnosis* (e.g., overlapping phenomenology with other disorders such as unipolar depression, borderline personality disorder and attention deficit disorder (Keramatian et al., 2022; Scott et al., 2021; Smith et al., 2024; Temes et al., 2024)); (3) *age of onset* (whether it can BD be diagnosed pre-pubertally (Duffy et al., 2020; Goldstein et al., 2017; Malhi and Bell, 2020; Malhi et al., 2023b)); (4) *healthcare challenges* (difficulties accessing services, lack of awareness and training of mental health professionals (Cotton et al., 2023, 2025)); (5) *stigma* (Keramatian and Morton, 2023); and (6) *individual* factors (Keramatian et al., 2022). Delayed diagnosis can be associated with harm due to inappropriate treatments (e.g., concerns about antidepressants and associated risks of switching into hypomania/mania), greater duration and severity of depressive symptoms, ultradian cycling, increased hospitalisations, treatment resistance, poor physical health, substance misuse, functional decline and deliberate self-harm and suicide (Altamura et al., 2010; Brady and Sonne, 1995; Gitlin, 2018; Kim et al., 2025; Nasrallah, 2015; Post et al., 2010; Preuss et al., 2021; Świtaj et al., 2025).

Treatments have their limitations. Despite lithium being the gold standard, its use has declined largely due to concerns about the complexity of its medical management, active marketing of alternatives and significant side effects (Malhi et al., 2023a). Clinicians are latterly prescribing atypical antipsychotics, antidepressants and anticonvulsants (e.g., sodium valproate, lamotrigine); however, these also have risks and limitations (Greil et al., 2024; Kessing, 2024; Malhi et al., 2023a; Singh et al., 2024). Polypharmacy is the norm, and it is unclear how to balance risk and benefits of multiple drug prescriptions (Bauer et al., 2018). Medications do not counteract depressive and subsyndromal symptoms, cognitive and functional impairments, and poor QoL (Bonnín et al., 2019; Gitlin and Miklowitz, 2017; Miklowitz and Gitlin, 2015). Medication non-adherence is common and associated with relapse, hospitalisation, suicidality and higher treatment costs (Jawad et al., 2018).

While pharmacotherapy is typically the first-line treatment, adjunctive psychosocial interventions can help manage acute depressive episodes and support long-term maintenance (Yatham et al., 2018). Efficacious psychosocial interventions, recommended in clinical practice guidelines (Murray et al., 2017; Yatham et al., 2018), include psychoeducation, cognitive behavioural therapy, family-focused therapy, and interpersonal and social rhythm therapy (Chatterton et al., 2017; Chiang et al., 2017; Miklowitz and Chung, 2016; Miklowitz et al., 2021; Rabelo et al., 2021; Steardo et al., 2020). But resource scarcity and costs, and lack of trained therapists, means that few receive them (Chiang and Miklowitz, 2023; Hajda et al., 2016). In the United Kingdom, only one in three individuals with BD has been offered psychotherapies and four in five had received no psychoeducation to assist with the disorder management (Bipolar UK, 2022). This is extremely disconcerting given that adjunctive psychosocial interventions can reduce time to remission, delay and reduce relapse, improve functional outcomes as well as address other issues such as medication adherence, emotion dysregulation, subthreshold symptoms and QoL (Azevedo et al., 2025; Murray et al., 2017; Novick and Swartz, 2019; Reinares et al., 2014).

Promising yet nascent evidence suggests that interventions delivered in the early stages of BD can mitigate poor long-term outcomes (Joyce et al., 2016; Post et al., 2010; Ratheesh et al., 2023; Vieta et al., 2018). However, a review of 14 international clinical guidelines found that there were few evidence-based recommendations for this stage of the disorder (Chia et al., 2019). Most pharmacological and psychosocial interventions have been designed and tested for adults with chronic disorder and neglect young people and those experiencing early stages of the disorder (Cotton et al., 2019; Ratheesh et al., 2023; Vallarino et al., 2015).

Burden, poor treatment at a community level and excess mortality underpin the urgent need for indicated prevention, early identification, facilitation of treatment access and more efficacious evidence-based treatments for BD (Ferrari et al., 2016).

## Challenges with research in BD

Underpinning the above challenges is the limited research investment in BD, especially compared to depression and psychotic disorders (Cotton et al., 2023). In the United Kingdom, for instance, BD receives only 1.5% of research funding that is allocated to mental health despite accounting for 17% of the disease burden attributable to mental health (MQ, 2021); such data need to be collated for Australia. Perpetuated by the competition for limited funds, there can be a lack of collaboration between research groups. Beyond funding limitations, research difficulties can be due to the disorder’s complexity, logistical issues (e.g., recruitment) and the broader research landscape.

Population identification and research recruitment can be difficult (Desai et al., 2021; Scholle et al., 2000). Because of BD’s complexity and diagnostic delays, there are many with undiagnosed/misdiagnosed BD who are overlooked for research (Cluss et al., 1999; Scholle et al., 2000; Scott et al., 2025; Strawbridge et al., 2023). Variable help-seeking and problems accessing services can also be challenging. UK research has indicated that a quarter of those with BD do not seek help from services (Humpston et al., 2021). Only 40% of those with a self-reported diagnosis had received mental health care in the previous 12 months, and only 16.9% had BD-specific care (Humpston et al., 2021). Most individuals with BD are treated at generalist services (Kessing et al., 2013; Vieta, 2011). Generalist services are difficult to access due to high symptom severity and significant acute risks being typical thresholds due to resource scarcity (Morriss, 2010; National Institute for Clinical Excellence, 2015). Such services do not cater to those with *episodic* difficulties; those who could benefit from treatment fall through the gaps (National Institute for Health and Care Excellence, 2014). Consequently, it is difficult to recruit large representative samples for clinical and epidemiological studies (Cluss et al., 1999; Scholle et al., 2000). Recruitment cannot rely on one clinical site or recruitment strategy; collaboration is required across services and groups to address such issues (Cotton et al., 2023, 2025).

There are also difficulties in conducting and translating treatment-related research. Many studies have strict inclusion/exclusion criteria which impede generalisability (Wong et al., 2018). Treatment non-adherence is common (20–60%) and can not only impact study outcomes but can also have long-term clinical and economic consequences (Hong et al., 2011). Placebo-controlled trials are ethically complex (Vieta and Carné, 2004). There can also be high placebo response rates (Keck et al., 2000). Most pharmacological randomised controlled trials (RCTs) last for 6–12 weeks; however, BD requires lifelong management. Standard RCTs take years to complete, and even if a treatment is found effective, rarely are treatments embedded into care (Frieden, 2017). Longitudinal studies are resource intensive and have their own barriers (McInnis et al., 2022). More flexible study designs such as adaptive, sequential multiple assignment randomised treatment (SMART) and basket trial designs might assist with resourcing and earlier translation of research findings (Bowden and Singh, 2015; Hawke et al., 2025; Jeon and Fava, 2015).

To advance BD research and to reduce diagnostic delays and improve treatments and outcomes for BD, there is an urgent need for greater involvement of all stakeholders (Cotton et al., 2023, 2025). Key stakeholders can be divided into four often overlapping categories, including ‘lived’ (individuals with lived/living experience of BD), ‘loved’ (carers/supporters of people with BD), ‘laboured’ (clinicians, service providers, policy makers) and ‘learned’ (researchers) (Killackey, 2023: Figure 1). These groups often operate in silos despite working towards the same purpose – better outcomes for those impacted by BD. As shown in other mental health fields (e.g., the Accelerating Medicines Partnership^®^ Schizophrenia [AMP-SCZ; Wannan et al., 2024]), by working collaboratively across stakeholder groups and with increased person power and momentum, there can be large studies informing innovation and advances to care.

**Figure 1.**
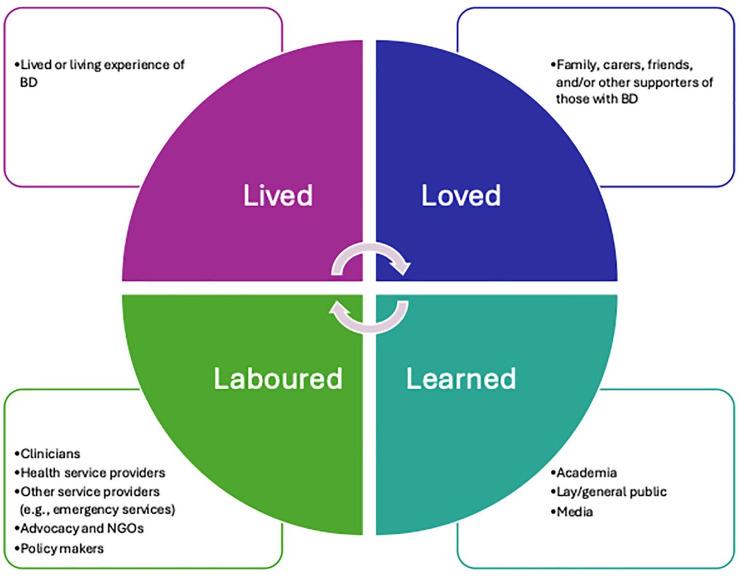
The four Ls framework adapted from Killackey (2023).

The other issue for BD and mental health research more broadly relates to delays in translation. There are significant delays in the application of new knowledge into practice, with some estimates of delays from ‘bench’ to ‘bedside’ being 17 years (Wallace, 2013). There are instances where invaluable knowledge and interventions never reach those who could benefit most (Wallace, 2013) – e.g., psychotherapies for BD (Chiang and Miklowitz, 2023). It is possible, however, to reduce these delays as demonstrated with responses to COVID-19 (Shukla et al., 2024).

### What are the solutions?

The field of early intervention for BD is nascent (Rowland and Marwaha, 2018; Vieta et al., 2018). We need a consolidated approach for indicated prevention and early intervention that embraces the complexity and heterogeneity of the disorder, addresses the need for collaboration across all key stakeholders and identifies earlier, effective and cost-effective approaches to translate ‘what works’ into real-world practice.

Work is being done internationally and nationally to address the barriers facing clinical practice and research into BD. The International Society of Bipolar Disorder (ISBD) has several task forces actively driving work in this area. ISBD task force publications relevant to early intervention have focused on psychological intervention precursors and prodromes (Faedda et al., 2019; Tremain and Murray, 2024), early intervention for BD (Ratheesh et al., 2023) and clinical staging (Kupka et al., 2021). The ISBD Taskforce on Early Intervention is currently leading an international Delphi Study covering research priority setting, evaluating intervention evidence and defining participant populations for future early intervention research. The BD2 initiative is investing in large scale cohort studies in the disorder (www.bipolardiscoveries.org/).

We have also been working with the Daymark Foundation, a Canadian family philanthropic trust, to identify the international barriers limiting the progression of early intervention for BD. This work has not only involved interviews with international experts (Cotton et al., 2023, 2025), but also has also entailed three in-person meetings, including a satellite meeting in the United States (2023, coinciding with the ISBD conference) and larger meetings in Switzerland (2023, coinciding with the International Early Intervention and Prevention in Mental Health Association [IEPA] Conference) and Iceland (2024, prior to the ISBD Conference). At the 2023 Daymark Foundation meeting in Switzerland, participants identified areas that they believed would have the greatest impact on early intervention for BD (Cotton et al., 2023). The priorities identified, in order of importance, included the development of an international data network, implementation of a staged care learning network, an international BD awareness campaign, building capacity in primary care, addressing the issue of measurement and consideration of lithium clinics (Cotton et al., 2023). Working groups have been formed to focus on addressing community awareness, at-risk states, measurement issues and the need for big data (Cotton et al., 2023, 2025).

There is also substantive work currently being undertaken in Australia through CORE-BD. CORE-BD aims to (1) support novel and quality collaborative research; (2) foster capacity building and workforce development; (3) nurture and expand partnerships; and (4) ensure effective knowledge translation.

## What is involved in CORE-BD?

### Research innovation

**Figure 2.**
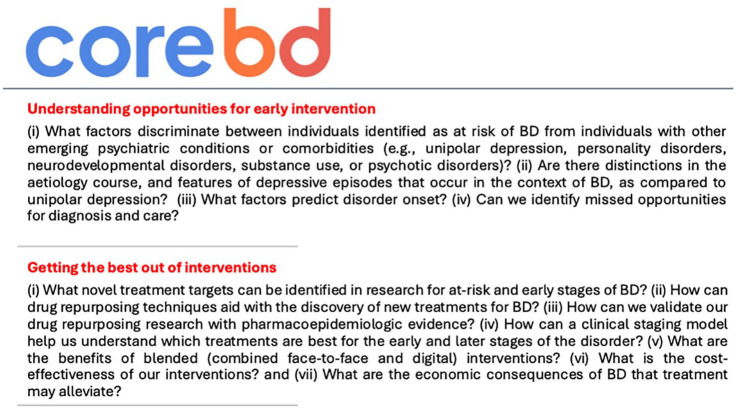
Two key themes underpinning CORE-BD and research questions of interest.

**Figure 3.**
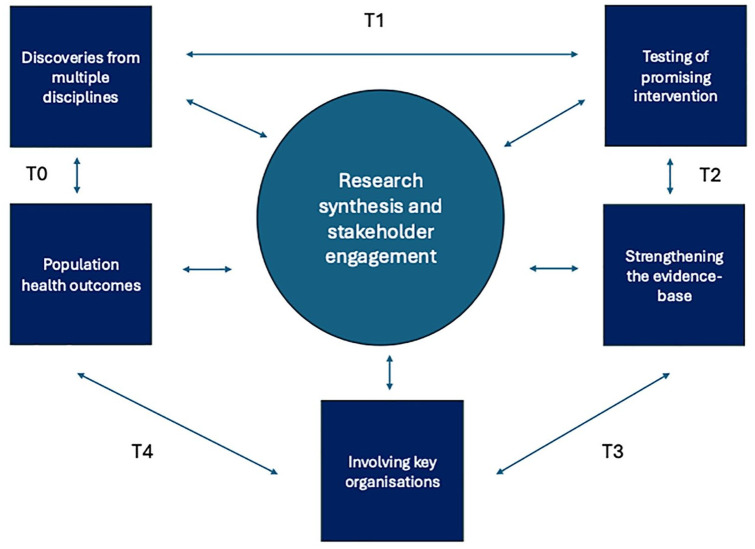
CORE-BD’s ‘T’ framework.

CORE-BD is structured around two main research themes: (1) advancing understanding of opportunities for indicated prevention and early intervention in individuals at risk or recently diagnosed with BD; and (2) addressing gaps in effective treatments through (a) treatment development and trial design and (b) economic evaluation to support implementation strategies. The research spans basic science to health economic methodologies and aims to address a wide range of questions (see Figure 2). The programme is informed by Milat and Li’s (2017) adaptation of the National Institutes of Health (NIH) translational research model (see Figure 3; Zerhouni, 2003).

### Importance of diverse representation

CORE-BD incorporates extensive stakeholder engagement. The *Brains Trust* comprising individuals with lived and/or loved experience and representatives from community-based advocacy groups, provides ongoing input to the research agenda, implementation, and translation.

A *Clinical and Academic Translation* group will (1) develop a dissemination plan, (2) provide guidance to clinical services on the implementation and (3) convene an annual symposium. A *Policy Think Tank* will use this combined expertise to support resource dissemination (e.g., assessment guidelines, decision tools, training materials) and engage with government stakeholders to promote system-level change. Economic analyses will underpin these efforts.

### Workforce development and capacity building

There are recognised gaps in clinical and research capacity (Fisher et al., 2018; Jenkins et al., 2011; Rakofsky and Dunlop, 2012; Stein et al., 2015). The *Education Committee* will explore approaches to address these training needs, including workshops for clinicians and students.

Research capacity building will focus on early- and mid-career researchers (EMCRs), a group facing barriers such as limited funding, scarce mentorship opportunities and few training pathways (Sperry et al., 2023). CORE-BD will align with the ISBD Early Mid-Career Committee (EMCC) (Douglas et al., 2024) and offer training, mentorship and collaborative opportunities. Scholarships and fellowships for postgraduate and postdoctoral researchers will include mentorship, interdisciplinary training, co-design skills development and involvement with advisory groups. In addition, a global *Student Network* and a planned *EMCR Network* will provide forums for collaboration, skill development and engagement with the broader BD research community.

### Other planned activities

CORE-BD will include systematic reviews and meta-analyses to synthesise evidence and inform practice. New analytic approaches – including computational modelling and machine learning – will be explored to better understand BD onset and progression (e.g., Glavatskiy et al., 2024). Findings will inform the development of resources for stakeholders, which will be shared via the project website and through targeted knowledge translation activities (e.g., workshops, presentations). Future directions include building partnerships and seeking competitive funding to expand and sustain research in this area. Co-design will remain central across all initiatives.

## Conclusion

The lack of research and clinical focus on BD to date, both generally and towards early intervention, has contributed to its often debilitating course, significant associated burden and many unmet needs, despite the fact it is an eminently treatable disorder. Apart from work being done internationally, CORE-BD provides the platform nationally to foster partnerships with key stakeholders and to develop co-designed frameworks to advance research, address the critical shortage of trained researchers and ensure that knowledge is translated to help those most at need. Through CORE-BD, various activities and opportunities will be available. We invite those interested to reach out and to monitor updates on our website (www.corebd.com.au). Investment of this kind has the potential to improve outcomes, not only for those in the early stages of BD, but also across the lifespan.
